# Deciphering the Implications of *Escherichia coli* in Inflammatory Bowel Disease: From Symbiont to Pathogen

**DOI:** 10.3390/pathogens15050548

**Published:** 2026-05-19

**Authors:** Gitana Maria Aceto, Katia Falasca, Desirèe Berardinucci, Ludovica Cavallo, Alessio Mangiò, Giuseppe Mancuso, Raffaella Muraro, Rachele Ciccocioppo, Teresa Catalano

**Affiliations:** 1 Department of Sciences, “G. d’Annunzio” University of Chieti-Pescara, 66100 Chieti, Italy; 2 Clinic of Infectious Diseases, Department of Medicine and Science of Aging, “G. d’Annunzio” University of Chieti-Pescara, 66100 Chieti, Italy; katia.falasca@unich.it; 3 Department of Sciences, Medical School Student, “G. d’Annunzio” University of Chieti-Pescara, 66100 Chieti, Italy; desiree.berardinucci@studenti.unich.it; 4 Department of Sciences, Chemistry and Pharmaceutical Technology Student, “G. d’Annunzio” University of Chieti-Pescara, 66100 Chieti, Italy; ludovica.cavallo@studenti.unich.it; 5 Department of Human Pathology, University of Messina, 98125 Messina, Italy; alessio.mangio@studenti.unime.it (A.M.); giuseppe.mancuso@unime.it (G.M.); 6 Department of Innovative Technologies in Medicine & Dentistry, University “G. d’Annunzio” of Chieti-Pescara, 66100 Chieti, Italy; raffaella.muraro@unich.it; 7 Department of Medicine and Ageing, “G. d’Annunzio” University of Chieti-Pescara, 66100 Chieti, Italy; rachele.ciccocioppo@unich.it; 8 Department of Clinical and Experimental Medicine, University of Messina, 98125 Messina, Italy

**Keywords:** inflammatory bowel disease, *Escherichia coli*, pathobionts, adherent-invasive *E. coli*, diffusely adherent *E. coli*, gut microbiota, host–microbe interactions, mucosal colonization, antimicrobial resistance, clinical management

## Abstract

Inflammatory bowel disease (IBD) is a chronic inflammatory condition resulting from complex interactions between the immune system, genetic predisposition, and the gut microbiota. In this context, *Escherichia coli* (*E. coli*) plays a dual role in the human gut, ranging from harmless commensal strains to pathobionts capable of promoting intestinal inflammation. A growing body of evidence suggests that specific *E. coli* pathotypes, such as adherent-invasive *E. coli* (AIEC) and diffusely adherent *E. coli* (DAEC), contribute to the development and progression of IBD. This narrative review critically examines the microbiological, immunological, and clinical evidence supporting the role of *E. coli* in IBD, with particular emphasis on mechanisms of mucosal colonization, host–microbe interactions, and persistence within the inflamed intestinal environment. Furthermore, the lack of a standardized operational definition and the limited reproducibility of the AIEC phenotype are addressed, as well as uncertainty about the role played by *E. coli* as a primary initiator of the disease or as an opportunistic amplifier of intestinal inflammation, and the varying strength of evidence supporting associations with Crohn’s disease versus ulcerative colitis. Diagnostic implications, antimicrobial resistance, and therapeutic aspects are addressed as downstream and context-dependent consequences of *E. coli*–host interactions, with relevance for disease management and therapeutic response in patients with established IBD. By integrating data from experimental models, clinical studies, and translational research, the review identifies areas of consensus, ongoing controversy, and major knowledge gaps in IBD pathophysiology and clinical practice.

## 1. Introduction

Inflammatory bowel disease (IBD), including Crohn’s disease (CD) and ulcerative colitis (UC), is a chronic inflammatory condition involving complex interactions between host immunity, genetic susceptibility, and gut microbiota. Growing evidence supports the concept that specific *E. coli* pathotypes act as intestinal pathobionts, contributing not only to disease persistence but also potentially to disease initiation under permissive host and environmental conditions. Within this framework, the relevance of *E. coli* to IBD pathophysiology is increasingly interpreted through its capacity to behave as a context-dependent pathobiont, whose contribution to disease emerges primarily under conditions of mucosal inflammation and immune dysregulation.

*E. coli* is an early and stable colonizer of the human gastrointestinal (GI) tract, where it predominantly exists as a commensal organism. In this ecological context, *E. coli* contributes to microbial homeostasis and host metabolism, producing essential metabolites and antimicrobial compounds [1,2]. Despite this beneficial role, its remarkable genomic and metabolic plasticity enables a transition toward opportunistic pathogenicity when mucosal integrity or immune surveillance is compromised [3,4]. This context dependent shift toward pathogenic behavior is frequently driven by the horizontal acquisition of virulence determinants and antimicrobial resistance (AMR) genes, fostering the emergence of highly virulent and multidrug-resistant lineages of increasing global health relevance. Within the intestinal niche, *E. coli* occupies a dynamic interface with the epithelium, shaping host–microbe interactions through integrated metabolic and signaling networks [5]. Inflammation, interspecies competition, dietary factors, and pharmacological pressures act as major selective forces, collectively promoting rapid genetic diversification and metabolic rewiring. These selective forces favor nutrient-scavenging strategies, including mucus utilization, and facilitate the emergence of opportunistic pathotypes under dysbiotic conditions [6]. In the context of IBD, these adaptive and evolutionary features are particularly relevant because chronic intestinal inflammation generates a selective environment that favors the emergence and persistence of *E. coli* strains with enhanced fitness in the inflamed mucosa. Both antibiotic and non-antibiotic drugs profoundly alter gut microbial community structure, with incompletely understood consequences for colonization resistance against enteropathogens [7,8]. *E. coli* readily acquires resistance to diverse xenobiotics (not only antibiotics but also preservatives and environmental pollutants) via efflux pump upregulation (notably AcrAB–TolC), altered membrane permeability, and plasmid mediated horizontal gene transfer (HGT). Co-selection processes further accelerate the expansion of multidrug-resistant (MDR) strains under overlapping selective pressures [9]. Through these processes, *E. coli* emerges as a central ecological mediator linking microbial community dynamics, epithelial barrier integrity, immune signaling, and metabolic homeostasis. Adaptive evolution in *E. coli* is strongly shaped by mutation rate dynamics. While bacterial mutation rates typically approximate 10^−3^ per genome per generation [10], mutator phenotypes frequently arise during intestinal colonization, particularly under inflammatory conditions [11]. These strains accelerate the acquisition of adaptive mutations, enabling rapid ecological optimization. *In vivo* studies in murine models demonstrate that *E. coli* undergoes rapid within-host evolution, often mediated by transposable element insertions in fitness-associated loci [12]. This ability for controlled mutability and metabolic flexibility enables *E. coli* to withstand oxidative, acid, osmotic, and thermal stresses, supporting survival and growth in hostile environments. Adaptive responses are governed by coordinated genomic and transcriptomic changes that facilitate the rapid emergence of resistance phenotypes and niche-specific traits [13,14,15]. Importantly, adaptive evolution often proceeds via clonal interference, resulting in soft selective sweeps characterized by the coexistence of multiple advantageous lineages rather than fixation of a single dominant clone [11]. This evolutionary behavior provides a useful framework for understanding the persistence of *E. coli* in inflamed intestines and the dissemination of AMR lineages. Resistance in commensal *E. coli* is widely recognized as an early ecological indicator of antibiotic selective pressure in human and animal populations [16]. While antimicrobial resistance in *E. coli* represents a global public-health concern, its relevance in IBD lies primarily in the way recurrent inflammation and repeated antibiotic exposure reshape intestinal microbial ecology, influence pathobiont persistence, and complicate clinical management.

Genetically, *E. coli* is structured into distinct phylogenetic groups (A, B1, B2, C, D, E, F, and G) and several cryptic clades (I–V), identified using conserved genetic markers such as *chuA* and *yjaA* [17]. These groups differ markedly in colonization efficiency and persistence, with B2 strains displaying enhanced long-term intestinal fitness [12]. These evolutionary dynamics provide a useful framework for understanding why genetically diverse *E. coli* lineages are repeatedly recovered from inflamed intestinal tissues rather than representing stable, disease-specific clones. Certain pathotypes have evolved specialized virulence strategies to subvert epithelial defenses. Enteropathogenic (EPEC) and enterohemorrhagic *E. coli* (EHEC) utilize a Type III secretion system (T3SS) to translocate effector proteins into host cells, inducing actin cytoskeletal remodeling and the formation of “attaching and effacing” lesions accompanied by microvillus effacement [18]. Dietary composition is a critical regulator of intestinal microbial ecology and epithelial function [19]. High-fat diets impair mitochondrial bioenergetics, disrupt epithelial hypoxia, and promote facultative anaerobe expansion within the gut [20,21,22,23]. Although these metabolic mechanisms are not specific to *E. coli*, they help explain why facultative anaerobes such as *E. coli* are selectively enriched in the inflamed intestinal environment characteristic of IBD.

**Figure 1:**
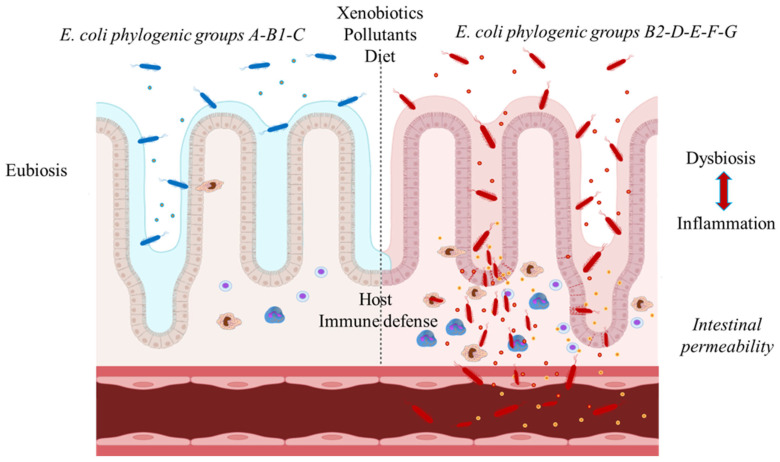
*E. coli* epithelial intestinal interactions—from symbiont to pathogen. The figure illustrates the loss of intestinal mucosal barrier capacity, also known as ‘leaky gut’. This is associated with an altered immune defense system, as well as with factors such as diet, environmental pollutants, foreign substances, oxidative stress, and an imbalance in the microbiota. Phylogroups A, B1 and C are typically harmless, but can become harmful. B2, D, E, F and G strains are extraintestinal pathogens (ExPEC) that are often associated with virulence.

Antibiotic exposure and intestinal pathophysiology are tightly interlinked. Antibiotic-driven disruption of the gut microbiota not only promotes the expansion of resistant *E. coli* populations but also compromises barrier integrity and fosters chronic inflammatory states, including IBD and colorectal cancer (CRC). Emerging evidence implicates iron-acquisition pathways as central regulators of virulence expression during commensal to pathobiont conversion [24] (Figure 1). Furthermore, the reciprocal interactions among diet, microbiota, and epithelial barrier function are increasingly recognized as core drivers of IBD and colitis-associated CRC pathogenesis [25]. Mechanistic and *in vivo* studies identify adherent-invasive *E. coli* (AIEC) as prototypical pathobionts in IBD, capable of exploiting inflammation-associated mucosal nutrients to enhance virulence and drive IL-1β/Th17-dependent CD pathology [26,27]. Conversely, selected strains such as *E. coli* Nissle 1917 exert anti-inflammatory effects and are employed as therapeutic probiotics. AIEC strains are predominantly associated with CD, whereas diffusely adherent *E. coli* (DAEC) is more commonly linked to UC, where mucosal colonization contributes to epithelial damage and sustained inflammation [28]. Despite increasing attention to *E. coli* pathotypes as potential contributors to IBD, fundamental challenges remain unresolved, including the absence of a standardized operational definition for AIEC, the resulting variability in experimental reproducibility, and uncertainty as to whether these bacteria act as primary drivers of disease or are selectively enriched as a consequence of intestinal inflammation.

Finally, the intestinal microbiota and associated biofilms play a contributory role in colorectal carcinogenesis and IBD through the production of reactive oxygen species (ROS), release of genotoxic metabolites, and amplification of inflammatory signaling cascades [29]. These processes culminate in dysbiosis, impaired immune surveillance, and altered nutrient metabolism [30]. Disease-associated microbiomes display expanded resistomes, underscoring the pervasive circulation of antibiotic resistance genes (ARGs) under selective pressures imposed by antibiotics and inflammation. Horizontal transfer of ARGs within the gut further amplifies resistance dissemination, while resistant bacteria and their metabolites may modulate inflammatory responses and reduce the efficacy of anticancer therapies [31]. Despite extensive characterization of *E. coli* as both a commensal organism and a pathobiont, the molecular and ecological determinants governing its context-dependent transition toward pathogenic behavior remain incompletely understood, particularly with regard to host metabolism, non-antibiotic selective pressures, and within-host evolutionary dynamics. A central challenge in this field is distinguishing correlation from causation and determining whether disease-associated *E. coli* represents stable, causative entities or context-dependent phenotypes selectively enriched under inflammatory and dysbiotic conditions. This unresolved distinction—whether *E. coli* acts as a primary initiator of intestinal inflammation or as an opportunistic responder to an already inflamed gut environment—has major implications for the interpretation of experimental findings, diagnostic strategies, and therapeutic targeting, and frames the ongoing debate addressed in this review.

### Methodology

This article provides a narrative and critical synthesis of current evidence on the roles of *E. coli* pathotypes in inflammatory bowel disease (IBD), integrating microbiological, immunological, clinical, diagnostic, antimicrobial resistance, and therapeutic perspectives. A comprehensive literature search was conducted in PubMed/MEDLINE without a strict temporal restriction while focusing primarily on publications from the past decade. Seminal earlier studies were included when conceptually relevant to contextualize pathogenic and mechanistic frameworks. The final literature update was performed on 30 April 2026. Only articles published in English were considered. The search strategy combined MeSH terms and free-text keywords (e.g., *E. coli*, AIEC, DAEC, pathobiont, IBD, gut microbiota, antimicrobial resistance), used alone or in combination. Representative Boolean search strategies included combinations such as (“*Escherichia coli*” AND “inflammatory bowel disease”), (“adherent-invasive *Escherichia coli*” OR “AIEC” AND “Crohn’s disease”), and (“diffusely adherent *Escherichia coli*” OR “DAEC” AND “ulcerative colitis”). Eligible sources included peer-reviewed experimental, clinical, genomic, metagenomic, and translational studies, as well as systematic reviews, meta-analyses, and international guidelines. Articles that were non-peer-reviewed, methodologically insufficient, purely descriptive, redundant, or not relevant to the pathophysiological, clinical, diagnostic, or therapeutic aspects addressed in this review were excluded. Given the narrative and integrative nature of this review, no formal quality-scoring system or systematic selection algorithm was applied. Instead, evidence was interpreted and hierarchized according to its conceptual relevance and level of biological and clinical inference. Human clinical and biopsy-based studies were prioritized whenever available, particularly those linking *E. coli* colonization or functional traits to disease phenotype or outcome. Experimental *in vitro* and animal model studies were incorporated to support mechanistic plausibility but are clearly distinguished from associative clinical evidence in humans. Selected studies were critically synthesized into thematic sections covering disease mechanisms, diagnostic approaches, antimicrobial resistance, and therapeutic implications, with particular emphasis on validated phenotypic, genetic, and functional criteria distinguishing commensal from pathogenic *E. coli* strains and on unresolved controversies and translational knowledge gaps.

## 2. Pathophysiological and Immune Interactions Between *E. coli* and the Host

*E. coli* is characterized by remarkable plasticity, which, as demonstrated primarily in experimental models, allows it to shift from a commensal organism to an opportunistic pathogen in the setting of impaired immune surveillance or mucosal barrier dysfunction. [3,4]. While these mechanisms provide strong biological plausibility for a role of *E. coli* in intestinal inflammation, their interpretation is complicated by the fact that most evidence derives from reductionist experimental models that do not fully capture the temporal and ecological complexity of human IBD. Accordingly, this section examines the mechanistic pathways through which *E. coli* has been proposed to contribute to the persistence and amplification of intestinal inflammation, with particular attention to the limitations of extrapolating experimental findings to human disease.

### 2.1. Microbiota and Epithelia Interplay

The intestinal epithelium represents a dynamic interface between the host immune system and the gut microbiota, where homeostatic interactions are continuously negotiated to maintain barrier integrity and immune tolerance. Under physiological conditions, this interplay relies on tightly regulated microbial metabolic activity, epithelial oxygen consumption, and immune signaling pathways that collectively preserve mucosal homeostasis. Disruption of this finely balanced system, through inflammation, environmental perturbations, or altered microbial community structure, can profoundly modify epithelial function and reshape host–microbe interactions.

In the context of IBD, such alterations are consistently associated with reduced microbial diversity and the relative expansion of facultative anaerobic bacteria, including *E. Coli*. Rather than acting in isolation, *E. coli* occupies a niche shaped by epithelial barrier dysfunction, altered nutrient availability, and immune dysregulation, which together influence its capacity to persist at the mucosal surface and interact with host tissues. The microbiota contributes to colonization resistance against invading pathogens by competing for metabolites, producing inhibitory substances and triggering protective immune responses. However, commensal bacteria can promote host resistance and immune-mediated protection, albeit with reduced tissue colonization capacity from pathogens. This depends on the production of interferon (IFN)-γ by innate cells and CD4+ T cells. As recently demonstrated, several members of the microbiota can prevent intestinal *Salmonella* infection by enhancing responses to IFN-γ [32]. Enteric Gram-negative bacteria, including *E. coli* or their components characterized by an outer membrane (OM) containing lipopolysaccharides (LPS), induce IFN-γ production by stimulating myeloid dendritic cells to release IL-12/IL-18, activating NK cells, ILC1s, and CD4+ T cells. This, along with IFN-β, amplifies macrophage bactericidal activity to control infection. *E. coli* antigens, particularly in IL-10 deficient environments, trigger high IFN-γ output [33,34]. Notably, *E. coli*–host interactions are not exclusively pro-inflammatory; adhesion of specific *E. coli* strains has been shown to promote IL-10 production, supporting immunoregulatory pathways that temper mucosal inflammation [35].

The maintenance of physiological epithelial hypoxia in the colon is increasingly recognized as a cooperative process involving both host metabolism and the resident microbiota. While butyrate-producing anaerobes play a central role by enhancing epithelial oxygen consumption and stabilizing hypoxia-inducible factor-1α (HIF-1α), evidence indicates that commensal *E. coli* also contributes to this homeostatic axis. Through respiration-dependent oxygen consumption at the mucosal surface, non-pathogenic *E. coli* can reinforce epithelial hypoxia, thereby supporting HIF-1α-driven barrier-protective gene expression [22]. In parallel, epithelial PPAR-γ signaling regulates mitochondrial activity and limits oxygen diffusion into the lumen; its disruption results in loss of physiological hypoxia and promotes dysbiotic expansion of *E. coli*. Conversely, restoration of PPAR-γ activity re-establishes epithelial hypoxia and constrains *E. coli* within a controlled commensal state [36]. Collectively, these findings support an integrated model in which microbial metabolites, bacterial respiration, and epithelial metabolic regulation converge to sustain HIF-1α stabilization, intestinal barrier integrity, and host–microbiota homeostasis.

Perturbation of the intestinal barrier disrupts epithelial integrity by impairing tight-junction assembly and mucus layer maintenance, notably via reduced occludin and mucin-2 expression, thereby increasing intestinal permeability. Barrier dysfunction fundamentally alters host–microbe interactions, enabling closer contact between luminal bacteria and the mucosal immune system. In this altered ecological context, commensal organisms endowed with specific adaptive traits may gain a selective advantage, favoring the expansion of pathobionts capable of persistent immune engagement and potential amplification of intestinal inflammation.

### 2.2. Leaky Gut and E. coli

Increased intestinal permeability, commonly referred to as “leaky gut”, represents a recurrent feature of IBD and reflects a loss of epithelial barrier integrity rather than a discrete pathogenic event. Barrier dysfunction facilitates enhanced exposure of the mucosal immune system to luminal microbes and microbial products, thereby reshaping host–microbe interactions and altering the ecological pressures that structure the intestinal microbiota. Within this permissive environment, facultative anaerobic bacteria such as *E. coli* are consistently enriched. Importantly, the expansion of *E. coli* under conditions of impaired barrier function does not imply a uniform pathogenic role, but rather highlights the context-dependent emergence of functionally distinct subpopulations. Among these, AIEC has been operationally defined by its capacity to adhere to intestinal epithelial cells, invade host tissues, and persist within immune cells in experimental settings. These traits have positioned AIEC as a prototypical pathobiont in Crohn’s disease, while also underscoring the challenge of distinguishing primary pathogenic drivers from phenotypes that are selectively favored by the inflamed and permeable intestinal niche. The environmental and metabolic factors enabling AIEC strains associated with CD to flourish in the ileum have been identified [37]. Within the context of pre-existing mucosal barrier dysfunction and immune dysregulation, these factors contribute to the establishment of a permissive niche for opportunistic bacteria, including pathogenic *E. coli*, thereby facilitating bacterial persistence, translocation, and, in susceptible hosts, systemic dissemination [38,39]. Such phenomena have been documented across a range of clinical conditions, including IBD, irritable bowel syndrome (IBS), obesity, metabolic syndrome, enteric infections, and prolonged antibiotic exposure, all of which are characterized by microbiota alterations and compromised intestinal barrier function.

While intestinal dysbiosis and the depletion of beneficial microbial taxa are well-recognized contributors to IBD exacerbation, increasing evidence indicates that specific gut microorganisms with pathogenic potential are enriched in affected individuals. These organisms, termed pathobionts, are thought to actively participate in disease initiation and perpetuation. Current hypotheses on IBD pathogenesis increasingly emphasize the disease-promoting activities of invasive *E. coli* strains [39]. In this context, key environmental and metabolic factors enabling AIEC strains to expand and persist within the inflamed ileal mucosa of patients with CD have been identified [26].

Numerous *in vitro* and *in vivo* studies have described functional traits associated with AIEC, such as mediated adhesion FimH and carcinoembryonic antigen-related cell adhesion 6 (CEACAM6), epithelial invasion, intracellular persistence in macrophages, and modulation of autophagy, that, in selected experimental settings, are associated with enhanced inflammatory responses. In murine models, AIEC strains have been reported to exacerbate experimental colitis or induce stronger inflammation than non-AIEC *E. coli* strains. However, the translational relevance of these findings is limited by strong model dependence, substantial intra-AIEC heterogeneity, and the context-dependent nature of the inflammatory phenotype, which appears to reflect specific host–microbiota–environment interactions rather than a fixed virulence property [40]. The expansion of *E. coli*, particularly AIEC, is frequently observed in IBD, especially CD. Experimental studies demonstrate that AIEC possess intrinsic pro-inflammatory properties, including epithelial adhesion and invasion and the capacity to survive and replicate within macrophages, thereby sustaining cytokine production and chronic inflammation [41,42]. However, intestinal inflammation also reshapes the gut milieu, promoting the persistence of facultative anaerobes such as *E. coli*. Consequently, AIEC are increasingly regarded as pathobionts whose expansion reflects both cause and consequence of mucosal inflammation, consistent with a bidirectional disease model [40]. Clinical evidence further supports this complexity, as antibiotic strategies targeting AIEC have failed to yield consistent clinical benefit despite bacterial clearance [43]. Notably, host immune mechanisms, including AIEC-specific IgA responses, can limit bacterial epithelial colonization, underscoring the importance of host–microbe interactions in disease outcome [44].

AIEC has been shown to preferentially colonize an inflamed and dysbiotic intestinal niche characterized by oxidative stress and the enrichment of *Enterococcus* species. Intestinal dysbiosis promotes the production of ROS through the activation of enzymes such as NADPH oxidases (NOX), thereby establishing a pro-inflammatory and oxidative microenvironment. In *E. coli*, tolerance to oxidative stress is mediated by a complex transcriptional regulatory network centered on the OxyR system, which responds primarily to hydrogen peroxide (H_2_O_2_), and the SoxRS system, which is activated in response to superoxide radicals (O_2_•^−^) [14]. These systems regulate the expression of antioxidant enzymes and pathways involved in ROS detoxification, as well as DNA repair mechanisms and core metabolic processes [14]. This transcriptionally driven metabolic remodeling is aimed at limiting endogenous ROS production and preserving cellular integrity under oxidative stress. In aerobic conditions, *E. coli* can decrease respiratory flux to reduce electron leakage from the respiratory chain, thereby limiting ROS generation and promoting a shift toward fermentative metabolism as a protective strategy. This process involves the modulation of NADH dehydrogenase activity and the cytochrome bo_3_ ubiquinol oxidase, accompanied by the diversion of carbon flux into fermentation pathways. These adaptive responses are coordinated by global regulatory systems, including ArcA/ArcB and FNR, which collectively restrain respiration-associated ROS production and enhance bacterial survival under oxidative stress [45]. These observations collectively support an ecological model in which inflammation-associated changes in the intestinal niche selectively favor the expansion of facultative anaerobes such as *E. coli*, raising the possibility that bacterial overgrowth may reflect adaptation to inflammation rather than primary disease initiation.

### 2.3. Major Groups of Pathogenic E. coli

To better understand the epidemiological relevance of pathogenic *E. coli* strains, a phylogenetic classification system based on the presence of specific genetic markers has been developed [46]. Recently, *E. coli* strains have been assigned to eight phylogroups, which differ in variable gene content, pathogenicity, antibiotic resistance, host relationship, and environmental factors. These eight phylogenetic groups belong to *E. coli sensu stricto* (A, B1, B2, C, D, E, F, G), while the other five phylogroups are considered cryptic *Escherichia* clades [47]. Clade I is linked with human diseases, whereas clades II to V could have environmental origins [48]. Group B2/D strains are typically extra-intestinal pathogens (ExPEC), while A and B1 are often commensal, though they can be pathogenic. Acute *E. coli* infections range from enteritis to community- or hospital-acquired UTIs, as well as septicemia, post-surgical peritonitis, and neonatal meningitis. They are generally not linked to chronic inflammatory bowel diseases because the strains involved are non-enteroinvasive and do not trigger the immune responses seen in CD or UC [49].

Intestinal biopsies and stool samples from individuals with IBD reveal a higher prevalence of *E. coli* strains from the B2 phylogenetic group. Evidence from cell-based experiments and animal studies indicates that these *E. coli* pathobionts exhibit pathogenic traits, suggesting that they may contribute to the development of IBD. In fact, the inflamed mucosa of UC provides oxygen and nutrients that can favor *E. coli* phylogroup B2, which often carries the genomic island *pks* encoding a hybrid polyketide synthase–non-ribosomal peptide synthetase (PKS–NRPS) system [50]. *E. coli* pks^+^ is an intestinal strain that synthesizes colibactin, a genotoxin causing DNA damage in colon cells. *E. coli pks^+^* is notably more numerous in UC patients than in healthy controls, as well as in CRC [51,52]. Indeed, *pks^+^ E. coli* does not invade deeply but damages epithelial DNA and promotes dysplasia and carcinogenesis more than chronic inflammation [47,53]. Although *pks^+^ E. coli* can be detected in CD, it is less consistently present than AIEC. Moreover, some patients harbor strains that exhibit both AIEC and pks^+^ features, which can exacerbate epithelial injury and macrophage activation. These observations have reinforced concerns about a potential link between UC, chronic intestinal inflammation, and an increased risk of CRC [50]. Although diarrheagenic strains are not associated with IBD, they induce inflammation and pathophysiological alterations in the intestine. The main *E. coli* pathotypes, key virulence factors, and validated mechanisms specifically associated with IBD are listed in Table 1. Several lines of evidence indicate that AIEC strains are particularly prevalent among CD-patients, while DAEC has been associated with UC [50,52]. AIEC can invade gut epithelial cells and survive in macrophages, with consequential tissue inflammation, whereas DAEC strains can fix to the rectal mucosa by adherence factors [52].

Despite well-characterized adhesion mechanisms and epithelial effects in experimental models, clinical evidence supporting a direct pathogenic role for DAEC in UC remains limited and less consistent than that reported for AIEC in CD.

### 2.4. AIEC

The evidence linking AIEC to intestinal inflammation primarily stems from *in vitro* systems and animal models. In contrast, clinical studies in humans primarily demonstrate associations with disease activity and phenotype rather than direct causality. AIEC is unusually abundant in the ileal mucosa of patients with CD [61]. The reference AIEC strain LF82 binds to ileal epithelial cells via type 1 pili adhesins (FimH), which interact with CEACAM6 receptors overexpressed in patients with CD. Sequencing analyses demonstrate that AIEC strains typically harbor recent amino-acid substitutions in the FimH adhesin, consistent with pathoadaptive evolution. These mutations enhance bacterial adhesion to CEACAM-expressing epithelial cells and are associated with increased pro-inflammatory responses in experimental models [55,62]. Although these findings support a role for FimH-mediated adhesion in promoting intestinal inflammation, a direct causal contribution of AIEC to disease onset in humans has not been conclusively demonstrated [55].

AIEC strains possess multiple mechanisms that facilitate interaction with the host intestinal immune system [63]. They can access the intestinal mucosa via outer membrane vesicles [64] and through the expression of the outer membrane protein A (OmpA), which induces glycoprotein-96 overexpression and promotes bacterial translocation into lymphoid tissues [65]. AIEC further exploit microfold (M) cells, which actively sample luminal antigens. This enables dissemination to the lamina propria and mesenteric lymph nodes.

AIEC also alter tight-junction organization and epithelial barrier integrity, increasing paracellular permeability and facilitating bacterial translocation across the intestinal epithelium [66,67]. Some *E. coli* strains expressing the type 1 pili adhesin FimH also harbor the *pks* genomic island encoding the genotoxin colibactin. These strains can adhere to the intestinal epithelium and deliver colibactin in close proximity to host cells, inducing DNA damage and thereby promoting genomic instability and colorectal carcinogenesis [67]. Experimental evidence suggests that *E. coli* producing colibactin are primarily associated with epithelial DNA damage, dysplasia, and tumorigenesis. However, their role in chronic mucosal inflammation is more variable and context-dependent. Conversely, it is believed that AIEC activity involves releasing immunostimulatory factors that activate mucosal immune responses and sustain chronic inflammation [68]. Although mucosal metabolites generally promote *E. coli* growth, it is ethanolamine that specifically enhances the expansion of AIEC, particularly when combined with inflammation-related amino acids, glutathione, and fucose, which is a sugar associated with symbiosis. AIEC exploit this metabolic flexibility through specialized microcompartments and stress response pathways. Specifically, ethanolamine and glutamine increase the motility, infectivity, and pro-inflammatory activity of AIEC. In IL10-deficient mice, ethanolamine metabolism is associated with inflammation, whereas fucose metabolism promotes symbiosis. Overall, AIEC bacteria have adapted to utilize host-derived metabolites, enabling a transition from commensal inhabitants to bacteria capable of amplifying intestinal inflammation under permissive conditions [26]. Indeed, AIEC are found in about 29% of CD patients, compared with 9% of healthy controls. In UC, AIEC are present in about 12% of patients and 5% of controls. Overall, UC patients have almost a threefold higher likelihood of carrying AIEC compared with controls (OR 2.82) [68]; moreover, a close association of AIEC phenotype strains with CD may also exist in pediatric patients [69]. Importantly, the AIEC phenotype encompasses a heterogeneous group of strains defined by functional assays rather than by conserved genetic markers, resulting in substantial inter-study variability and limited reproducibility. This heterogeneity complicates comparison across cohorts and may partly explain inconsistencies in clinical and interventional studies.

### 2.5. DAEC

In contrast to CD, UC is characterized by inflammation largely confined to the colonic mucosa, where epithelial barrier dysfunction and altered host–microbe interactions play a central role. Within this anatomical and immunological context, *E. coli* strains exhibiting a diffusely adherent phenotype (DAEC) have been reported with increased frequency in patients with UC, particularly during active disease. As with AIEC in CD, the association between DAEC and UC does not imply a uniform or causative pathogenic role. Rather, DAEC represents a functional adhesion phenotype defined by its capacity to adhere diffusely to intestinal epithelial cells, predominantly through Afa/Dr family adhesins, in experimental settings. The emergence and persistence of DAEC in UC are best interpreted within a context-dependent framework, in which epithelial barrier impairment, mucosal immune activation, and changes in the colonic microenvironment collectively shape selective pressures favoring specific bacterial traits. In patients affected by UC, the adhesion of the DAEC pathotype to the colonic epithelium is mediated by the interaction of fimbriae with the cell membrane receptors [70]. DAEC strains harboring the Afa/Dr family of adhesins (Afa/Dr+) contain specific fimbrial adhesins, such as Dr and F1845 [56]. DAEC also includes *E. coli* strains harboring AfaE-I, AfaE-II, AfaE-III, AfaE-V, Dr-II, and NFA-I adhesins. Afa/Dr-positive strains express *afa*, *dra* and *daa* operons that encode Afa/Dr adhesins, whereas Afa/Dr−DAEC strains expressing AIDA-I adhesin represent a subgroup of atypical EPEC [71]. AFaE-III, Dr, and F1845 bind to the human decay-accelerating factor (hDAF, CD55), carcinoembryogenic antigen (CEA), CEACAM1, and CEACAM6 cell receptors, whereas NFA-1 adhesin binds to CEA, AfaE1 and Dr-II adhesins bind to hDAF but not to CEACAMs, and the Dr adhesin also binds to type IV collagen [71,72]. Bacteria expressing Afa/Dr adhesins can act on differentiated epithelial cells, triggering cytoskeletal rearrangements involving F-actin, followed by microvilli loss and epithelial dysfunction [70]. Accordingly, adherence of Afa/Dr strains to polarized epithelial cells through hDAF, CEACAMs, and CEA structurally and functionally alters the epithelial barrier by increasing permeability [71]. The secreted autotransporter toxin (SAT) produced by DAEC further disrupts tight-junction integrity and increases epithelial permeability [73]. DAEC adhesion also induces IL-8 production, promoting the recruitment of polymorphonuclear leukocytes, TNF-α, and IL-1β, thereby contributing to persistent intestinal inflammation [71]. Despite well-characterized adhesion mechanisms and epithelial effects, clinical evidence supporting a direct causal role for DAEC in UC remains limited and less consistent than that reported for AIEC in CD.

### 2.6. Interactions Between the Host and the Pathobiont E. coli

Inflammatory bowel disease is characterized by profound disruptions in gut homeostasis, including microbial imbalance, loss of the protective mucus layer, and increased intestinal permeability. These changes activate pro-inflammatory Th1 and Th17 pathways through heightened antigen presentation by dysfunctional dendritic cells. This dual activation is strongly linked to chronic intestinal inflammation. Pathogenic *E. coli*, particularly AIEC strains, do activate pro-inflammatory Th17 cells and can promote Th1-like responses, including hybrid Th1/17 populations [74]. Nutrient metabolism by some AIECs may impact intestinal homeostasis through nutrient metabolism [27]. Indeed, AIEC strains that possess the PduC enzyme, which is necessary for utilizing 1,2-propanediol derived from fucose fermentation, appear to be becoming more prevalent in patients with Crohn’s disease. These strains promote an inflammatory response in T lymphocytes. Furthermore, CX3CR1^+^ intestinal immune cells (mononuclear phagocytes, or MNPs) are crucial for AIEC-induced Th17 cell and IL-1β production in mouse models, resulting in inflammatory colitis. This process requires PduC’s enzymatic activity to generate propionate, a metabolite that stimulates MNPs to produce IL-1β together with bacterial LPS [27]. The process reflects a self-sustaining cycle of innate immune dysregulation that drives IBD pathology. In the intestine, the main players in innate immunity are macrophages, which exhibit remarkable plasticity, responding to various signals to maintain the composition of the microbiota and tissue microenvironment homeostasis. They prevent bacteria that breach the epithelial barrier from spreading throughout the body, and their immune responses to luminal and adherent bacteria restrict the growth of pathogens and pathobionts [75]. AIEC strains evade effective phagocytic killing and are able to replicate within macrophages, driving sustained TNF-α secretion and chronic inflammation. Their ability to survive inside macrophages under environmental stress (such as fluctuations in pH) further modifies macrophage function, ultimately impairing their phagocytic capacity [41,76,77]. CX3CR1^+^ macrophages shift toward an M1 inflammatory phenotype, promoting chronic inflammation, extracellular matrix buildup, and fibrosis. Innate lymphoid cells (especially NKp44^−^ ILC3) produce elevated IL-17 and IL-22, worsening epithelial injury and recruiting additional immune cells [78,79]. Genetic variants identified through genome-wide association studies (GWAS) in individuals with IBD converge on pathways that regulate intracellular bacterial handling, creating a favorable environment for AIEC. Genomic and functional studies demonstrate that AIEC strains selectively accumulate in genetically susceptible hosts and exhibit disease-specific adaptations [68,80]. Moreover, *CARD9*, an adapter gene located downstream of pattern recognition receptors (PRRs), has been linked to an increased risk of IBD. It plays a crucial role in maintaining mitochondrial homeostasis in neutrophils. The absence of CARD9 disrupts the regulation of oxidative metabolism, resulting in the hyperactivation of the electron transport chain and the accumulation of mitochondrial reactive oxygen species (mtROS), as well as the premature activation of intrinsic apoptotic pathways. This increases vulnerability to oxidative stress, reducing neutrophil longevity and impairing their ability to kill fungi, degranulate, and release NETs. Insufficient containment of microbial pathogens, particularly opportunistic fungi, exacerbates the mucosal inflammatory response and promotes the progression of chronic intestinal inflammation [81,82]. Neutrophils can intensify inflammation through a process called suicidal NETosis, which depends on the NADPH oxidase-driven production of ROS. During this pathway, activated neutrophils release web-like structures composed of DNA and antimicrobial proteins, known as neutrophil extracellular traps (NETs) [83]. This form of NETosis typically leads to neutrophil death within 2–4 hours after activation. Excessive or dysregulated NET formation can amplify tissue damage and sustain inflammatory responses. Neutrophil extracellular traps accumulate in the intestinal mucosa of patients with CD and UC, where they intensify tissue injury and perpetuate chronic inflammation [84]. Following antibiotic and AIEC-associated intestinal dysbiosis, an increased ability of neutrophils to form web-like structures was observed, suggesting a possible overgrowth of immune-activating intestinal pathobionts [85]. Intestinal dysbiosis was also associated with an increased ROS production *in vitro* by cultured gut bacteria, resulting in elevated oxidative stress in the colon and inflammation [85]. *E. coli* isolated from patients with CD or UC display distinctive genomic features that support their persistence in the inflamed intestinal environment. These include genes involved in managing oxidative stress, enhanced iron acquisition systems, and metabolic adaptations that allow for survival under inflammatory conditions. Many strains also harbor antibiotic-resistance plasmids, likely acquired through horizontal gene transfer, which further contribute to their fitness in the dysbiotic gut. These strains also exhibit specialized adaptations to the inflamed intestinal mucosa, characterized by high-affinity adhesion to and active invasion of epithelial cells, the capacity to persist and replicate within macrophages by resisting phagolysosomal killing, and the metabolic ability to exploit inflammation-derived substrates (including ethanolamine and host-generated nitrate) as alternative nutrient and electron sources that enhance their competitive fitness in the dysbiotic gut [41,42,76].

The plasmids, often carrying extended-spectrum beta-lactamases (ESBL), allow for horizontal gene transfer in environments like wastewater and the gut, significantly increasing the resistance of recipient strains [86,87] (see Table 2).

The contribution of AIEC to intestinal inflammation is driven by its capacity to persist within the host, a process that critically depends on the integrity of innate immune defenses. Once they have entered intestinal cells, AIEC opportunistically exploit any alterations in innate immunity and autophagy mechanisms. These processes, critical for recognizing, confining, and eliminating intracellular microorganisms, are often compromised in individuals with genetic variants associated with IBD, such as those involving nucleotide-binding oligomerization domain-containing protein 2 (NOD2), also known as caspase recruitment domain-containing protein 15 (CARD15), autophagy related 16 like 1 (ATG16L1), and immunity related GTPase M (IRGM). When these pathways are impaired, xenophagy, a specialized form of autophagy, is unable to effectively degrade bacteria already in the cytoplasm, creating a permissive environment in which AIEC can survive, replicate, and stimulate a persistent inflammatory response. In this context, the interplay between host genetic vulnerability and pathogenic characteristics of AIEC becomes a central element in the progression and chronicity of intestinal inflammation. *E. coli* (particularly AIEC) interferes with autophagy and endolysosomal maturation [63]. Variants of the *NOD2* gene associated with fibrostenosing CD and suppression of IL-10 transcription underscore the pivotal role of IL-10 in maintaining gut mucosal immune homeostasis [88]. NOD2 functions as a cytosolic receptor for muramyl dipeptide, mediating bacterial sensing and contributing to innate immune responses and tolerance toward the commensal microbiota. Loss-of-function *NOD2* variants represent a key pathogenic mechanism in CD, resulting in impaired bacterial clearance and exaggerated inflammatory responses [89]. Beyond microbial recognition, NOD2 plays a critical role in autophagy and immune regulation, including the modulation of adaptive immunity. Consistently, CD-associated variants in *NOD2*, *ATG16L1*, and *IRGM* converge on defective antimicrobial autophagy, compromising the control of intracellular pathogens. Specifically, the *NOD2* mutations R702W, G908R, and 1007fs impair the muramyl dipeptide-induced signaling required for recruitment of the autophagy machinery, thereby limiting the initiation of xenophagy. More broadly, dissecting the interplay between genetic susceptibility and host–microbial interactions may explain why only a subset of genetically predisposed individuals develop IBD [90]. The T300A variant in *ATG16L1*, in turn, destabilizes the ATG5–ATG12–ATG16L1 complex and impairs autophagosome formation, reducing the ability of intestinal cells to degrade invasive microorganisms. Finally, *IRGM* regulatory variants alter gene expression levels and interfere with autophagosome maturation and fusion with lysosomes, a crucial step in the degradation of microbial contents. Together, these defects affect complementary phases of the autophagic response, creating an environment permissive for the survival of intracellular bacteria—including AIEC strains—and thus contributing to the persistence of inflammation typical of IBD [91,92,93].

### 2.7. E. coli and CRC

Certain intestinal strains of *E. coli* have been implicated in the initiation and progression of CRC through the use of specific virulence factors and the activation of immune-inflammatory pathways [94]. The populations of *E. coli* adhering to the mucosa are significantly enriched in biopsy samples from CRC patients compared to healthy controls [95]. These strains show the ability to persist and replicate within macrophages, inducing a sustained pro-inflammatory response. Chronic exposure to inflammatory mediators promotes a microenvironment conducive to increased cell proliferation and tumorigenesis. Analysis of *E. coli* associated with mucosa in colon cancer and diverticulosis samples shows that 86% of cyclomodulin-positive strains belong to phylogroup B2 and often possess the *pks* genetic island, which produces colibactin, and/or *cnf* (cytotoxic necrotizing factor) genes. Although these B2 strains exhibit poor adherence and invasion *in vitro*, they can still induce CEACAM6 expression in intestinal epithelial cells, in a manner similar to strains associated with CD. *In vivo* experiments using CEACAM6-expressing mice demonstrate that the B2 11G5 strain, isolated from a colonic tumor, persists in the intestine for a prolonged period. This results in colon inflammation, epithelial damage, and increased cell proliferation [94]. A particularly important factor is the production of the genotoxin colibactin by most mucosa-associated bacterial strains of isolated from patients with CRC. These strains induce double-strand DNA breaks, arrest the cell cycle, and cause genomic instability and chromosomal rearrangements in eukaryotic cells. They have also been associated with carcinogenic effects in animal models. Furthermore, certain EPEC strains can cause chronic intracellular infections in colonic epithelial cells. This increases susceptibility to CRC by downregulating mismatch repair (MMR) proteins, which are responsible for DNA repair [96].

## 3. *E. coli* Pathobionts in Inflammatory Bowel Disease

The incidence of IBD is increasing globally, while its etiology remains largely undefined, although current evidence suggests a multifactorial pathogenesis. In this context, *E. coli* has received particular attention because certain strains, notably AIEC, exhibit pathobiont behavior [97]. Other *E. coli* pathotypes, including EAEC, DAEC, and EPEC, have also been associated with disease severity and clinical outcomes in certain patient groups [28].

The pathogenesis of IBD has increased, while its etiology remains largely unknown, although current evidence suggests a multifactorial pathogenesis.

### 3.1. Clinicopathological Impact of E. coli Pathotypes on IBD

A reduced microbial diversity accompanied by the expansion of facultative anaerobic bacteria with pro-inflammatory potential, including *E. coli*, is a well-established feature of IBD [98]. Among the *E. coli* pathotypes, AIEC are more prevalent in CD than UC and display key pathogenic features, including the invasion of intestinal epithelial cells, survival and replication within macrophages, and induction of TNFα-associated inflammatory responses [97]. Although experimental studies in animal models of colitis provide strong evidence supporting the pathogenic potential of AIEC under controlled conditions, validation of a direct causal role in human disease that fulfills Koch’s postulates remains limited [42]. Beyond AIEC, additional *E. coli* pathotypes, including EAEC, DAEC, and EPEC strains, have been implicated in modulating disease severity or clinical course in subsets of patients with IBD [28]. Importantly, host–*E. coli* interactions are not uniformly pro-inflammatory. Adhesion of specific *E. coli* strains has been shown to promote IL-10 production, suggesting the engagement of immunoregulatory pathways capable of attenuating mucosal inflammation [35]. In UC, the contribution of *E. coli* appears more heterogeneous. The prevalence of AIEC is intermediate between that observed in CD and in healthy individuals (UC: 35.7%, CD: 55.0%, healthy controls: 21.4%) and comparable to that reported in patients with colonic-restricted CD (40.0%) [99]; however, a definitive pathogenic role in UC has not been established. Experimental evidence primarily implicates *E. coli* in early disease stages, during which mucus layer disruption coincides with reduced microbial richness and increased *E. coli* abundance. In this context, diminished O-glycosylation of MUC2 has been linked to enhanced *E. coli* virulence via the activation of NF-κB signaling in epithelial cells, thereby contributing to epithelial barrier dysfunction [100]. Conversely, under specific conditions, *E. coli* may exert protective effects by limiting oxidative stress through iron sequestration and hydrogen peroxide detoxification, mechanisms potentially relevant to inflammatory resolution and maintenance of remission [101].

Among the non-AIEC pathotypes, increasing attention has focused on atypical enteropathogenic *E. coli* (aEPEC), which harbors the locus of enterocyte effacement pathogenicity island encoding a type III secretion system and intimin but lack Shiga toxin and classical adherence factors characteristic of typical EPEC strains [102]. Unlike typical EPEC, which are strongly associated with acute diarrhea, aEPEC are frequently detected in asymptomatic individuals and display prolonged persistence. Their pathogenic potential appears to depend on strain-specific effector repertoires and host susceptibility. Notably, aEPEC isolates derived from patients with IBD exhibit enhanced biofilm formation under aerobic conditions, whereas isolates from healthy controls preferentially form biofilms under anaerobic conditions, suggesting differential adaptation to inflammatory and dysbiotic environments. Whether aEPEC colonization precedes disease exacerbation or represents a consequence of mucosal inflammation remains unresolved; however, persistent colonization has been proposed to contribute to microbiota instability, low-grade inflammation, and diarrhea in UC. Collectively, these findings highlight the complex and context-dependent roles of *E. coli* pathotypes in IBD pathogenesis [99].

### 3.2. E. coli Association with IBD Severity and Clinical Management Challenges

A growing body of evidence supports a strong association between AIEC colonization and increased intestinal permeability, heightened inflammatory activity, and an elevated risk of IBD recurrence. This association links AIEC colonization to more severe disease phenotypes. However, it is unclear whether AIEC acts as a primary driver or is a consequence of intestinal inflammation [103]. In contrast, the association between DAEC and UC is weak and insufficiently demonstrated. The ecological and pathogenic profiles of *E. coli* differ consistently between CD and UC, reflecting adaptation to distinct inflammatory niches. In ileal Crohn’s disease, AIEC strains adhere to and invade intestinal epithelial cells. They also survive and replicate within macrophages and persist in deeper tissue compartments. This reflects the transmural nature of Crohn’s inflammation [5]. Genomic analyses of the reference AIEC strain LF82 have revealed the acquisition of multiple pathoadaptive genes and mutations derived from ExPEC B2 strains, as well as horizontal gene transfer from *Salmonella* and *Yersinia*, which collectively enhance bacterial virulence and may contribute to more aggressive and persistent disease behavior [104]. In UC, *E. coli* associations are more heterogeneous and less clearly linked to invasive phenotypes. The disease is primarily associated with B2 phylogroup strains harboring the *pks* genomic island. This island encodes the genotoxin colibactin, which induces DNA damage and an increased risk of CRC. Furthermore, *E. coli* strains associated with UC often exhibit increased resistance to host antimicrobial peptides, such as LL-37, which may facilitate their persistence in inflamed tissues [60,99]. Comparative genomic and functional studies indicate that UC selectively favors mucus-adapted, glycan-utilizing B2 pathobionts enriched in sialidase and mucin-degrading metabolic pathways. This confers a growth advantage in superficial, mucus-rich colonic inflammation [105,106,107]. These traits promote mucosal persistence rather than deep tissue invasion and are often coupled with ExPEC-associated virulence factors such as α-hemolysin, which contribute to epithelial barrier disruption and exacerbate colitis in susceptible hosts [108]. Together, these findings support a disease-specific ecological model in which UC selects for mucus-adapted pathobionts, whereas CD favors invasive, intracellularly persistent *E. coli* strains. Clinical studies further reinforce the link between *E. coli* pathotypes and disease severity. In a cohort of Egyptian IBD patients, high AIEC abundance in ileal biopsies was associated with CD, while phylogroups B2 and D correlated with more severe disease manifestations [103]. An increased prevalence of enteroaggregative *E. coli* capable of forming biofilms on the colonic mucosa of both UC and CD patients was also observed, suggesting a role in sustaining chronic inflammation [103]. These effects are compounded by antimicrobial resistance: qualitative antibiograms demonstrated widespread production of extended-spectrum β-lactamases and carbapenemases, and subsequent analyses revealed a direct correlation between antibiotic resistance and disease severity [109]. Genomic studies have further shown that *E. coli* isolates from IBD patients rapidly acquire additional resistance following antibiotic exposure [105]. Metagenomic profiling of the intestinal resistome confirmed a marked enrichment of antimicrobial resistance genes in UC, with *E. coli* representing the dominant reservoir. Detected serotypes included STEC, AIEC, ETEC, EPEC, and EHEC, underscoring the adaptive capacity of *E. coli* within the inflamed gut ecosystem [110]. The accumulation of resistance-associated and pathogenicity-related mutations likely contributes to adverse clinical outcomes, rendering IBD patients more susceptible to infections with multidrug-resistant organisms that further worsen disease severity and prognosis [111].

Beyond direct pathogenicity, *E. coli* also contributes to the diagnostic complexity of IBD management. A major clinical challenge is the accurate differentiation between an inflammatory disease flare and a superimposed infectious colitis, as endoscopic and histological findings often overlap [112]. Among IBD patients, *Clostridioides difficile* infection represents the most frequently identified superimposed infection and is consistently associated with more severe disease courses and poorer outcomes [113]. However, a broader range of gastrointestinal pathogens can be detected in symptomatic patients, supporting the routine use of extensive, multiplex PCR-based diagnostic panels. Recent evidence further suggests disease-specific patterns of infectious burden. An American study demonstrated distinct distributions of enteric pathogens among symptomatic patients with CD, UC, and non-IBD controls [114]. *Norovirus* and *Campylobacter* species predominated in CD, whereas UC was more frequently associated with bacterial pathogens, including *Campylobacter*, *Plesiomonas*, and *E. coli* [112]. These findings raise the possibility that pathogen-specific interactions with inflamed intestinal niches may differentially exacerbate disease activity depending on IBD subtype.

Finally, immunosuppressive therapies, particularly systemic corticosteroids, increase patients’ risk of opportunistic viral reactivations, such as those caused by *Cytomegalovirus* (CMV) and *Epstein–Barr virus* (EBV). These secondary infections can cause a superimposed viral colitis whose clinical, endoscopic, and histological features closely mimic those of an IBD flare, making accurate diagnosis challenging without quantitative PCR analyses performed directly on mucosal biopsy specimens [115]. Collectively, these observations underscore the multifaceted role of *E. coli* in modulating disease severity, complicating clinical assessment, and influencing outcomes in patients with IBD.

### 3.3. Diagnostic and Therapeutic Implications in IBD Patients

One of the cornerstones in the diagnosis and longitudinal monitoring of IBD is the measurement of fecal calprotectin, a calcium- and zinc-binding protein released by activated neutrophils that reliably reflects intestinal inflammatory activity. Beyond its established role as a biomarker, calprotectin also exerts direct antimicrobial effects through metal chelation, thereby limiting the availability of essential micronutrients to pathogenic bacteria [116]. In this context, Meheissen et al. reported a significant enrichment of *E. coli* phylogroup B2 in fecal samples with particularly high calprotectin levels, linking microbial composition to inflammatory burden [103]. Additional diagnostic tools include serological antibodies directed against microbial and host antigens, such as perinuclear anti-neutrophil cytoplasmic antibodies (pANCA), anti-Saccharomyces cerevisiae antibodies (ASCA IgG and IgA), antibodies against *E. coli* outer membrane porin C (anti-OmpC), and CBir1 flagellin [117]. Large cohort studies, including data from China, indicate that pANCA displays higher sensitivity in moderate to severe UC, whereas ASCA is more sensitive for ileal CD. Anti-OmpC antibodies may further assist in the differential diagnosis between intestinal tuberculosis and CD, where titers appear significantly higher in CD. Seropositivity against CBir1 is less common in colonic CD, underscoring disease-specific immune signatures [118]. Collectively, these serological profiles differ between CD and UC and are thought to reflect distinct patterns of host–microbial interaction. Notably, circulating antibodies (including ASCA, ANCA, anti-I2, anti-OmpC, and anti-CBir1) have also been associated with extra-intestinal manifestations, suggesting a broader systemic impact of microbial-driven immune activation.

From a therapeutic perspective, IBD remains a chronic, incurable condition in which current pharmacological strategies are aimed at inducing and maintaining remission, defined by mucosal healing, preservation of functional capacity, and improved quality of life. However, therapeutic response is often unpredictable, not only between different patients but also within the same patient over time. This variability has highlighted the need for personalized approaches to IBD management. In this setting, intestinal dysbiosis (previously discussed as a driver of disease severity) also emerges as a key determinant of therapeutic efficacy. Increasing evidence indicates that gut microbiota composition can influence both drug metabolism and clinical response, giving rise to the field of pharmacomicrobiomics. This discipline explores bidirectional interactions between medications and the gut microbiome and holds promise for future precision-medicine strategies. For example, the microbiota of the upper small intestine (duodenum and jejunum) plays a relevant role in the metabolism of orally administered drugs such as prednisolone and azathioprine, whereas colonic and fecal microbiota are critical for the efficacy of rectally administered agents such as budesonide and mesalamine [119].

The relationship between *E. coli* and IBD therapy is particularly illustrative. Sulfasalazine, a prodrug composed of mesalamine and sulfapyridine, requires bacterial azoreductase activity for activation and is metabolized by intestinal bacteria including *Enterococcus faecalis*, *E. coli*, and *Bacillus subtilis*. Dysbiosis can therefore influence drug activation, while sulfasalazine itself may reshape microbial communities. Accordingly, mesalamine therapy has been associated with the partial restoration of microbiota composition in UC and with reduced AIEC abundance in CD.

Among the immunomodulators, azathioprine and methotrexate are also influenced by microbial metabolism. Azathioprine can be converted to its active thioguanine nucleotides (6-TGNs) by intestinal bacteria such as *E. coli* and *Bacteroides fragilis*, which are often enriched in IBD patients [120]. Conversely, azathioprine administration can shift microbiota composition toward a less dysbiotic profile. *In vitro* studies further suggest that azathioprine at high concentrations may inhibit the growth of *E. coli* and *B. fragilis* and reduce the motility and virulence of AIEC, potentially attenuating inflammatory responses in CD [121]. These observations support the concept that AIEC represents not only a pathogenic contributor but also a potential therapeutic target.

Among the microbiota-based therapeutic strategies, the non-pathogenic *E. coli* strain Nissle 1917 (EcN) has shown efficacy in maintaining remission in distal UC. Originally isolated by Alfred Nissle in 1917, EcN exerts its effects through multiple mechanisms, including direct antimicrobial activity, competitive exclusion of enteroadhesive strains, and down-modulation of pro-inflammatory signaling pathways [122]. EcN is currently used as a microbial medicinal product in several gastrointestinal conditions, highlighting the translational potential of targeted microbiota interventions.

Overall, the integration of microbial biomarkers, host immune signatures, and microbiota–drug interactions represents a rapidly evolving area in IBD research. Although substantial progress has been made, the use of microbiota components as diagnostic markers or therapeutic tools remains an unmet clinical need and continues to drive intensive research efforts worldwide [123].

## 4. Diagnostic Methodologies for Pathogenic *E. coli* Strains in IBD

Diagnostic approaches for *E. coli* in IBD can be organized along a gradient of clinical applicability, reflecting a fundamental conceptual constraint inherent to the field. These approaches range from routine clinical diagnostics, which enable species-level identification but do not capture pathogenic phenotypes, to specialized translational assays and exploratory research tools. The latter are required to operationally define adherent-invasive *E. coli* (AIEC) as an etiological contributor to IBD within a functional pathobiont framework. At present, these approaches remain primarily research tools rather than validated diagnostic assays, reflecting ongoing challenges in standardization, reproducibility, and clinical interpretation. A critical challenge in diagnosing *E. coli*-associated IBD is the nature of AIEC itself. Unlike classical diarrheagenic *E. coli* pathotypes, AIEC does not represent a standardized taxonomic, serological, or molecular entity. Instead, it is defined by a set of functional properties, most notably adhesion to and invasion of intestinal epithelial cells and intracellular survival within macrophages, assessed through non-standardized *in vitro* assays. To date, no unique genetic marker or molecular signature has been shown to be either necessary or sufficient to define the AIEC phenotype. This intrinsic limitation constrains the clinical applicability of available diagnostic approaches and should guide the interpretation of all routine, translational, and advanced methodologies discussed below.

Rapid diagnosis of *E. coli* infections remains essential for effective clinical management of both intestinal and extraintestinal disease [63]; however, in the context of IBD, such findings must be interpreted in light of the functional and non-standardized nature of AIEC. Diagnostic strategies for pathogenic *E. coli* traditionally aim to detect the bacterium itself, its toxins, or specific virulence genes. Based on genetic and clinical criteria, *E. coli* strains are classified into three major groups: commensal strains lacking specialized virulence factors, intestinal pathogenic (diarrheagenic) strains, and extraintestinal pathogenic strains [28]. In the context of IBD, accurate identification of *E. coli* is particularly important to distinguish pathogenic variants from commensal members of the gut microbiota, as some strains act as pathobionts rather than classical pathogens [28]. Pathogenic *E. coli* strains employ a wide range of virulence and colonization factors that affect key host cellular functions and enable adhesion to and/or invasion of host cells [63]. Therefore, integrated diagnostic approaches are required to differentiate AIEC or DAEC from commensal or other pathogenic strains and to elucidate the molecular mechanisms underlying IBD pathogenesis. Identification of AIEC typically involves functional phenotypic assays, such as adhesion and invasion tests using epithelial cell lines and intracellular survival assays in macrophages, together with serological methods and molecular analyses targeting putative genetic markers, including virulence genes such as *fimH* [28,63]. Overall, the detection of IBD-associated *E. coli* relies on a combination of techniques that assess bacterial presence, behavior, and pathogenic potential, encompassing routine clinical diagnostics, translational methodologies, and exploratory research tools.

### 4.1. Routine Clinical Methods

Routine clinical methods are the first step in the diagnostic workflow; they enable species-level identification and the exclusion of classical diarrheagenic *E. coli* pathotypes. However, these methods cannot discriminate between functional phenotypes, such as AIEC or DAEC.

Culture-based methods use selective and differential media, such as eosin methylene blue (EMB) agar or MacConkey agar, to detect and isolate *E. coli* and “coliform” and to distinguish bacteria-based metabolic characteristics, respectively. Since *E. coli* has the intrinsic ability to ferment lactose and produce indole, it is identified by selective culture media prior to molecular testing [124]. Therefore, stool specimens were cultured in MacConkey agar plates to isolate Gram-negative enteric bacteria and differentiate lactose nonfermenting (colorless) from lactose-fermenting *E. coli* strains, like AIEC and DAEC, as well as to evaluate indole production [124,125]. CHROMagar™ STEC (CHROMagar™, Paris, France) is a medium used to distinguish *E. coli* O157:H7 from *E. coli* non-O157, containing substrates specifically recognized by β-d-galactosidase and β-d-glucuronidase. β-d-Galactosidase is produced by all *E. coli* strains, whereas β-d-glucuronidase is produced by all *E. coli* strains, except for non-sorbitol-fermenting (NSF) STEC O157:H7. This medium also contains selective agents, such as antibiotics [124]. 

Biochemical tests are required to differentiate *E. coli* from other bacteria and recognize different pathogenic *E. coli* subtypes. These tests do not distinguish between AIEC or DAEC pathotypes, which in turn differ in genetic virulence factors and, for AIEC, in tissue adhesion/invasion mechanisms and protease secretion. Using the IMViC test, *E. coli* results are positive in the indole and methyl red (MR) tests, and negative in the Voges–Proskauer (VP) and citrate tests [126]. The IMViC test represents a preliminary assay to separate *E. coli* from other genera, such as *Klebsiella* or *Enterobacter*, whereas molecular or cellular assays are needed to confirm the specific pathotype. The 4-methylumbelliferyl-β-D-glucuronide (MUG) test is used to assess β-glucuronidase activity, enabling rapid confirmation of typical *E. coli* strains and differentiation from MUG-negative variants such as O157:H7 [127]. Other analyses have been developed, such as Analytical Profile Index 20E (API 20E) systems (bioMérieux, Marcy-l'Étoile, France) that include biochemical tests to distinguish different members of the *Enterobacteriaceae* based on the enzymatic fermentation of sugars [126].

Antimicrobial susceptibility testing includes disk diffusion (Kirby–Bauer), that evaluates the bacterial growth on agar plates as susceptible, intermediate, or resistant; broth microdilution to determine minimum inhibitory concentrations (MICs) that prevent bacterial development; the rapid automated systems like VITEK^®^ 2 (bioMérieux, Marcy-l'Étoile, France) to identify resistant or sensitive profiles for many antibiotics.

Serotyping of *E. coli* relies on immunoassays detecting highly variable surface antigens, including the O (somatic), H (flagellar), and when present, K (capsular) antigens. The O antigen corresponds to the polysaccharide side chain of lipopolysaccharide (LPS) in the outer membrane and exhibits extensive structural variability, resulting in more than 180 distinct O serogroups [128]. Certain O:H combinations are strongly associated with specific pathotypes, such as O157:H7 with EHEC/STEC and O26:H11 with non O157 STEC [129]. In contrast, AIEC strains do not correspond to a single characteristic serotype and instead display heterogeneous O:H profiles, including O83:H1, O2:H6 and O6:H1, reflecting their genetic diversity [130]. Genes involved in O antigen biosynthesis are typically organized in a cluster located between the housekeeping genes *galF* and *gnd*, with most O antigens synthesized through the Wzx/Wzy pathway [128]. Agglutination based serotyping may be affected by cross reactivity between related serotypes, representing a recognized limitation of this approach [131].

Proteomic identification using matrix assisted laser desorption/ionization time of flight (MALDI TOF) mass spectrometry is now widely implemented in routine clinical microbiology laboratories. MALDI TOF enables the rapid and accurate identification of *E. coli* from pure cultures based on species specific protein spectra and is commonly applied following isolation and biochemical characterization, although discrimination between closely related species such as *E. coli* and *Shigella* may require specialized databases [132]. However, while MALDI-TOF MS provides rapid and reliable species-level identification from pure cultures, it does not allow for discrimination between pathogenic pathotypes, does not assess virulence or functional traits, and may show limited resolution for closely related taxa such as *E. coli* and *Shigella* without specialized databases.

### 4.2. Translational Methods

The genetic characterization of *E. coli* relies on nucleic acid-based techniques that detect virulence-associated genes that define the major diarrheagenic *E. coli* (DEC) pathotypes. These pathotypes include enteroinvasive *E. coli* (EIEC), which encompasses *Shigella* species based on shared virulence traits [131]. In the context of inflammatory bowel disease, these approaches facilitate the targeted investigation of pathogenic determinants. Although molecular methods are highly effective in defining classical DEC pathotypes by detecting specific virulence genes, they play an inherently indirect and supportive role in identifying AIEC because no molecular marker uniquely associates with the AIEC phenotype. DAEC target genes, such as *afa/dra*, should be analyzed after the completion of biochemical tests [133], while *chuA*, *fitA*, and *eefC* genes may have potential use as markers to discriminate AIEC strains from other *E. coli* pathovars [134].

PCR is used to recognize and confirm the presence of specific *E. coli* strains or serotypes after they have been identified using selective culture media [124]. Recently, non-O157:H7 EHEC strains not fermenting sorbitol have been identified by PCR. ETEC is detected by the amplification of *lt* and *st* genes. EPEC is identified by the pEAF plasmid or its encoded BFP factor. EAEC is identified through the AggR regulon, a transcriptional regulator that controls the expression of key virulence genes encoded on the plasmid of aggregative adherence (*pAA*) in EAEC [135]. EHEC/STEC is identified by nucleic acid amplification tests (NAATs) targeting Shiga toxin 1 (Stx1) and Shiga toxin 2 (Stx2). PCR also detects conserved regions of the thermolabile toxin gene (LT-A). EIEC is also detected via NAAT, and many EIEC strains are identified by the presence of the *lacY* gene, which encodes lactose permease [125].

*E. coli* isolates were tested by PCR to detect the six adherence genes (*afaE-1*, *afaE-2*, *afaE-3*, *afaE-5*, *daaE* and *aida/aah*) for the identification of DAEC [72]. Segura and colleagues examined all DAEC isolates by PCR to find six virulence associated genes (*sat*, *pet*, *sigA*, *pic*, *astA and fimH*) using appropriate specific primers [133]. AIEC needed *in vitro* adhesion/invasion testing while PCR for specific genes revealed that only 14% of CD-related AIEC harbor genes encoding Afa/Dr family adhesins [99]. Abdelhalim and colleagues demonstrated that all AIEC and non-AIEC strains from CD harbored the *fimH* gene, considered a major virulence factor and responsible for bacterial adherence and invasion, while the outer membrane hemin *chuA* gene (involved in iron uptake) and the ribosome association toxin *ratA* gene (affecting the 70S ribosome association and inhibiting protein synthesis) were the most common genes in both CD and healthy patients. All AIEC strains were negative for other invasion-associated genes [136].

Multiplex PCR-based methods enable the simultaneous detection of multiple genes in a single reaction [137]. This analysis was used for the phylogenetic group characterization, allowing *E. coli* strains to be classified into phylogroups known for their virulence, like B2 and D, which are more common in IBD patients. Multiplex PCR was also used to evaluate the presence of virulence-associated genes, including *fimA* (type-1 fimbriae), *draA* (Dr haemagglutinin), *neuB* (capsule K1), and *kfiC* (capsule K5) in AIEC strains [99].

The Biofire^®^ FilmArray^®^ GI Panel system (bioMérieux, Marcy-l'Étoile, France) is a fully automated multiplex PCR that detects *E. coli* through specialized molecular panels to identify the main pathogens responsible for infectious diarrhea (multiple bacteria, viruses, and parasites) as well as different *E. coli* pathotypes and pathogenic strains directly from stool samples, but does not include AIEC and DAEC [138].

Quantitative real-time PCR (RT-qPCR) is used to directly quantify bacterial load in clinical samples, including *E. coli* populations harboring genetic markers associated with DEC pathotypes or AIEC-related traits [139,140].

This method is also used to detect genes related to cell invasion and adhesion such as *fimH* in AIEC and *daaD* in DAEC, respectively [130,140]. *daaD* was selected since it was the best conserved gene related to the Dr adhesin phenotype [141]. MeltArray *E. coli* serotyping (*EST*) assay is a highly multiplexed, real-time PCR scheme that can simultaneously distinguish the five major DEC pathotypes and all available O and H antigen groups in a single test [131].

PCR is widely used to detect antibiotic resistance genes (ARGs) in *E. coli*, with the aim of elucidating the molecular mechanisms underlying this major public health concern. In clinical and research settings, phenotypic antimicrobial susceptibility testing is typically performed first, as it directly evaluates resistance at the functional level and guides subsequent molecular or genomic analyses. However, the relationship between genotype and phenotype is not always straightforward. Gene expression in *E. coli* is a conditional and hierarchical process in which the mere presence of a gene in the genotype does not guarantee its transcription or phenotypic manifestation. Transcription is tightly regulated by complex regulatory networks involving global and local transcription factors as well as alternative sigma factors that direct RNA polymerase to specific promoters, often with additional modulation by small RNAs and epigenetic mechanisms [142,143]. Consequently, gene expression and derived phenotypes are activated only in response to defined environmental cues, physiological states, or metabolic demands. This regulatory logic also applies to antibiotic resistance. The presence of antibiotic resistance genes does not necessarily result in phenotypic resistance, since many resistance determinants are weakly expressed, transcriptionally repressed, or conditionally activated, and may only be induced in the presence of antibiotics or under specific stress conditions [143,144]. As a result, substantial genetic variation within resistance gene families may remain clinically silent or uncatalogued, complicating genotype–phenotype correlations [144]. In this context, heteroresistance is common and is often misidentified or entirely missed in routine clinical susceptibility testing. Importantly, several negative effects on patient outcomes have been associated with infections caused by heteroresistant *E. coli* strains, including diagnostic failure and inappropriate antimicrobial therapy [145].

In addition to nucleic acid-based approaches, serological protein microarrays have emerged as a translational tool for the identification of disease-associated immune responses to *E. coli*. Protein arrays encompassing the entire *E. coli* proteome enable the detection of antibodies directed against specific bacterial proteins, including Era, YbaN, FocA, YcdG (RutG), and YhgN. These antibody signatures allow for discrimination between CD, UC, and healthy individuals, with reported sensitivity and specificity exceeding 80%, outperforming traditional serological markers such as ASCA and pANCA. Although not currently implemented in routine diagnostics, protein microarrays provide valuable insights into host–microbe interactions and support disease stratification in inflammatory bowel disease [146]. Overall, these findings highlight the potential of serological protein microarrays as a translational approach that bridges microbial profiling and host immune response, offering complementary information to nucleic acid-based diagnostics and providing a promising framework for improved patient stratification and personalized disease management in IBD.

### 4.3. Exploratory Research Tools

Since AIEC is defined by functional properties rather than by standardized molecular markers, its identification and characterization currently rely on exploratory research tools that examine bacterial behavior, host interaction, and genomic context. These tools include phenotypic testing, whole genome sequencing (WGS)-based multilocus sequence typing (MLST), CRISPR interference (CRISPRi), S1-nuclease pulsed-field gel electrophoresis (PFGE), as well as alternative emerging techniques and clinical metagenomics approaches.

The ability of *E. coli* to invade intestinal epithelial cells and replicate inside macrophages, a hallmark of AIEC associated with CD, or to predominantly interact with the epithelial surface, as described for DAEC in UC, can be assessed by phenotypic assays. These include the gentamicin protection assay to identify the ability of AIEC to invade gut epithelial cells [147]; adhesion and invasion assays on Caco-2 cells; and survival assays assessing intracellular replication within macrophages [136]. Adhesion assays for DAEC allow for the evaluation of a diffuse adherence pattern on HEp-2 or HeLa cells [148].

To monitor the global dissemination of highly resistant *E. coli* lineages, including strains associated with intestinal inflammation, sequence-type (ST) classification is performed using WGS-MLST, which provides improved accuracy compared with traditional PCR-based approaches. The application of MLST to *E. coli* is well-established, with numerous studies demonstrating its effectiveness in defining sequence types and monitoring the worldwide spread of multidrug-resistant lineages such as ST131 [149,150,151]. Essential resistance-associated genes in *E. coli* can be identified through CRISPRi screening, which systematically silences individual genes to determine those required for bacterial survival under antibiotic pressure, thereby revealing novel targets such as *degP* and *yacG* [152].

PFGE is performed to determine the degree of genetic variability and the antibiotic resistance profile among *E. coli* strains obtained from intestinal biopsies of CD patients and healthy controls [136]. In addition, plasmid profiling allows for the identification of the number, size, and types of plasmids present in a bacterial isolate, using approaches such as conjugation or S1-nuclease PFGE to track mobile genetic elements involved in the horizontal transfer of antibiotic resistance genes [153].

Activity-based probes, designed as chemiluminescent or fluorescent substrates for the colibactin-activating enzyme ClbP, enable the rapid analytical detection of *pks^+^ E. coli* by being cleaved upon enzyme activity, which triggers a measurable light signal and allows for the identification of *pks^+^* bacteria in stool sample resuspensions within one hour [154].

Although approaches based on gold nanoparticles are primarily suited for the detection of well-defined serotypes such as *E. coli* O157:H7 rather than AIEC, they exemplify emerging ultra-sensitive analytical strategies applicable to *E. coli* strain detection.

Gold nanoparticles (Au NPs) can be employed for the highly sensitive identification and quantitative detection of specific *E. coli* strains or serotypes by inductively coupled plasma mass spectrometry (ICP-MS), using monoclonal antibodies directed against defined surface antigens, such as those of *E. coli* O157:H7. In this system, signal intensity correlates with bacterial concentration, enabling sensitive analytical detection rather than functional or phenotypic characterization [155].

Some alternative techniques to PCR have been developed for the detection of multidrug-resistant *E. coli* strains, such as loop-mediated isothermal amplification (LAMP). LAMP rapidly and sensitively amplifies DNA at a constant temperature without a thermal cycler, making it faster and simpler than PCR and more tolerant to inhibitors commonly found in biological fluids such as blood and urine [156,157]. DNA sequencing of complex samples (feces, intestinal material, environmental matrices) can be performed through clinical metagenomics approaches, which enable the reconstruction of genomes and the identification of strain-level variants of *E. coli* directly from intestinal samples, allowing for the discrimination of different pathotypes and the recovery of complete bacterial genomes through the generation of metagenome-assembled genomes (MAGs), while functional metagenomics screens DNA fragments in a susceptible host such as *E. coli* to identify genes that confer a survival advantage under antibiotic pressure [158].

In summary, exploratory research tools provide mechanistic, functional, and evolutionary insights into *E. coli* pathogenicity and antimicrobial resistance that cannot be captured by routine or translational diagnostic approaches alone. While whole-genome sequencing enables high-resolution characterization when pure isolates are available, MAGs allow for the reconstruction of strain-level genomic frameworks directly from complex samples, and functional metagenomics facilitates the discovery of novel resistance determinants and adaptive traits beyond sequence homology. Collectively, these approaches expand the understanding of host–microbe interactions and bacterial evolution, laying the groundwork for the development of future translational biomarkers and targeted therapeutic strategies.

## 5. *E. coli* in Intra-Abdominal Infections: Antimicrobial Resistance Mechanisms and Pathogenic Determinants

In patients with IBD, intra-abdominal *E. coli* infections are more likely to progress to severe forms, making timely and appropriate clinical management particularly critical for patient outcomes and therapeutic decision-making [28,159]. *Escherichia coli* plays a central role in clinical practice as one of the most common causative agents of infectious diseases, particularly those involving the gastrointestinal tract. While many strains belong to the normal intestinal microbiota, pathogenic variants are capable of causing a wide spectrum of conditions ranging from localized intra-abdominal infections to severe systemic complications, including sepsis and septic shock, which are associated with high morbidity and mortality. The introduction and widespread use of effective antimicrobial therapies have significantly improved patient outcomes; however, their extensive and sometimes inappropriate application has contributed to the rapid emergence and dissemination of AMR. Consequently, a careful balance between the prompt initiation of adequate antimicrobial treatment and antimicrobial stewardship strategies is essential in order to preserve therapeutic efficacy and limit the selection of resistant strains. AIEC strains, and to a lesser extent DAEC, are capable of acquiring antimicrobial resistance predominantly through horizontal gene transfer. Conjugative plasmids represent the principal vehicles of resistance dissemination, frequently harboring genes encoding extended-spectrum β-lactamases such as *blaCTX-M*, *blaTEM*, and *blaSHV*, along with determinants conferring resistance to aminoglycosides (e.g., *aac*, *aad*), tetracyclines (*tetA/B*), and sulfonamides (*sul1/2*) [160,161]. Transposable elements, including *Tn3*- and *Tn21*-like transposons, as well as class 1 integrons, play a key role in the capture, accumulation, and rearrangement of multiple resistance cassettes, thereby promoting the emergence of multidrug-resistant phenotypes. In parallel, resistance may arise through chromosomal mutations, particularly in antibiotic target genes such as *gyrA* and *parC*, leading to fluoroquinolone resistance, or in regulatory pathways affecting outer-membrane porins and membrane permeability [162]. Overexpression of multidrug efflux systems, most notably the AcrAB-TolC pump, further contributes to reduced intracellular antibiotic concentrations and diminished antimicrobial activity [163]. Under antibiotic selective pressure, *E. coli* can activate the SOS response, an inducible stress pathway that increases mutation rates through the expression of error-prone DNA polymerases, thereby accelerating the adaptive evolution of resistance. In addition, exposure to bactericidal antibiotics may induce oxidative stress responses mediated by OxyR and SoxRS and intersect with SOS regulon activation. Several antimicrobial classes, including tetracyclines, chloramphenicol, and fluoroquinolones, have also been shown to upregulate efflux pump expression, further lowering intracellular drug accumulation. Importantly, in selected AIEC lineages, biofilm formation within the intestinal mucosa or on peritoneal surfaces enhances antibiotic tolerance and facilitates horizontal gene exchange, contributing to both phenotypic persistence and genotypic resistance [164,165,166]. These mechanisms collectively underscore the dynamic and multifactorial nature of AMR development in *E. coli*.

*E. coli* is consistently identified as the predominant pathogen in intra-abdominal infections (IAIs), with reported isolation rates ranging from approximately 36% to 43% [167]. The clinical management of these infections has become increasingly complex due to the global rise of MDR isolates, which significantly restrict empirical therapeutic options and are associated with delayed appropriate therapy and worse clinical outcomes. This challenge is particularly pronounced in complicated intra-abdominal infections (cIAIs), where polymicrobial involvement, high bacterial burden, and compromised host defenses converge.

From a pathophysiological perspective, abdominal *E. coli* infections should not be regarded solely as localized infectious events but rather as the result of a complex interplay between bacterial virulence factors, disruption of mucosal barrier integrity, microbial translocation, and the host immune response. Extraintestinal pathogenic *E. coli* (ExPEC) strains are especially adept at exploiting these conditions, leading to clinical manifestations that may rapidly progress to life-threatening scenarios, including severe sepsis and septic shock [168,169,170]. Host-related factors, such as immunosuppression, advanced age, metabolic comorbidities, and prior antibiotic exposure, further modulate disease severity and therapeutic response [171].

Complicated intra-abdominal infections caused by *E. coli* are most frequently sustained by ExPEC lineages. Unlike commensal intestinal strains, ExPEC isolates harbor a broad and coordinated repertoire of virulence determinants that enable colonization, immune evasion, and persistence in normally sterile extraintestinal sites. These include adhesins (e.g., type 1 and P fimbriae) facilitating attachment to host tissues, iron-acquisition systems such as siderophores (aerobactin, enterobactin, yersiniabactin), protective polysaccharide capsules, invasins, and toxins that contribute to tissue damage and systemic inflammation. The frequent co-localization of virulence and resistance determinants on mobile genetic elements in ExPEC strains further enhances their pathogenic potential, creating high-risk clones that are both highly virulent and difficult to eradicate therapeutically [172,173,174]. This convergence of virulence and AMR represents a critical concern in the management of abdominal infections and highlights the need for integrated microbiological surveillance and targeted therapeutic strategies.

### Clinical Impact, Source Control, and Antimicrobial Management of Intra-Abdominal E. coli Infections in IBD

In patients with IBD, intra-abdominal sepsis caused by *E. coli* frequently presents with early cardiovascular dysfunction, predominantly characterized by distributive shock resulting from inflammatory mediator-induced vasodilation. This vasoplegic state may progress to septic cardiomyopathy, further impairing tissue perfusion and amplifying multiorgan failure. Respiratory complications are common, as *E. coli*-driven cytokine release increases pulmonary capillary permeability and promotes the development of acute respiratory distress syndrome. Systemic activation of coagulation pathways may lead to sepsis-associated coagulopathy, and in severe cases, disseminated intravascular coagulation, exacerbating microvascular dysfunction and tissue hypoxia. Acute kidney injury and sepsis-associated encephalopathy are likewise prevalent, reflecting the combined effects of hypoperfusion, endotoxinemia, and inflammatory microcirculatory derangements, all of which significantly worsen prognosis in this vulnerable population [175]. Within this clinical setting, effective management of complicated intra-abdominal infections due to *E. coli* in IBD relies critically on timely and adequate source control. Antimicrobial therapy alone is insufficient in the presence of ongoing peritoneal contamination or uncontrolled infectious foci, and delays in surgical or percutaneous intervention are strongly associated with persistent organ dysfunction and adverse outcomes. In hemodynamically unstable patients with severe sepsis or septic shock, a damage control surgery strategy allows for rapid containment of contamination while limiting physiological stress, with definitive repair deferred until clinical stabilization. Conversely, in selected IBD patients with well-localized *E. coli* abscesses and no diffuse peritonitis, image-guided percutaneous drainage represents the preferred approach, reducing systemic inflammatory burden and potentially avoiding immediate surgery. Optimal source control therefore requires individualized, multidisciplinary decision-making based on disease severity, anatomical findings, and host factors [176]. Antimicrobial management must be equally tailored. Patients with IBD are frequently exposed to healthcare settings and antibiotics, increasing the likelihood of infection with multidrug-resistant *E. coli*, including ESBL-producing, and less commonly, carbapenem-resistant strains. While standard broad-spectrum regimens remain appropriate for community-acquired infections without resistance risk factors, hospital-acquired infections or severe presentations require early escalation to agents with reliable activity against ESBL-producing *E. coli*, with carbapenems remaining the mainstay in high-risk or septic patients [159]. The emergence of carbapenem-resistant *E. coli* further complicates management and necessitates the use of targeted agents such as ceftazidime/avibactam (with aztreonam when indicated), meropenem/vaborbactam, or cefiderocol, selected according to resistance mechanisms and susceptibility profiles [170,177,178].

## 6. Emerging Therapeutic Strategies and Future Directions in the Management of MDR *E. coli* Infections

Recent years have witnessed growing interest in innovative therapeutic strategies aimed at overcoming the limitations of conventional antibiotics in the management of *E. coli* infections, particularly in the context of MDR and antibiotic-induced microbiota dysbiosis. The global spread of antimicrobial resistance, coupled with the stagnation of the antibiotic development pipeline, has highlighted the urgent need for alternative or adjunctive approaches that extend beyond traditional bactericidal strategies [179,180,181].

Microbiome modulation has emerged as a promising avenue, as the restoration of gut microbial balance may reduce colonization by pathogenic strains and enhance colonization resistance against MDR organisms. Therapeutic approaches such as probiotics, prebiotics, and fecal microbiota transplantation (FMT) have demonstrated efficacy in selected clinical settings, most notably in recurrent *Clostridioides difficile* infection, where robust evidence supports their clinical use [182,183]. However, outside these specific indications, the role of microbiome-based interventions remains uncertain. In particular, current ESCMID and IDSA guidelines do not recommend microbiota modulation strategies for the treatment of intra-abdominal infections or sepsis, owing to limited and heterogeneous clinical evidence, unresolved safety concerns, lack of standardization, and challenges in patient selection [184]. Consequently, while microbiome-targeted therapies hold conceptual appeal, their clinical application in severe systemic infections remains investigational.

Bacteriophage therapy has re-emerged as a targeted antimicrobial strategy with the potential to selectively eliminate pathogenic *E. coli* while preserving the commensal microbiota. Advances in molecular biology and microbiome research have renewed interest in phages as precision antimicrobials, particularly in the setting of MDR and extensively drug-resistant (XDR) infections [185,186]. Preclinical studies and early clinical experiences, including compassionate-use cases and small series, have reported encouraging outcomes, especially where no effective antibiotic options remain [187,188]. Nevertheless, significant obstacles limit widespread clinical adoption, including regulatory and manufacturing challenges, the potential emergence of phage resistance, narrow host specificity, and the need for personalized phage cocktails and optimized delivery systems. Accordingly, current European Society of Clinical Microbiology and Infectious Diseases (ESCMID) and Infectious Diseases Society of America (IDSA) positions restrict phage therapy to experimental frameworks or compassionate use, pending results from well-designed randomized clinical trials [189].

Anti-virulence strategies represent another emerging therapeutic paradigm, focusing on the inhibition of bacterial pathogenic mechanisms rather than direct bacterial killing. By targeting virulence determinants such as adhesins, toxins, iron acquisition systems, or quorum-sensing pathways, these approaches aim to attenuate infection severity while reducing selective pressure for resistance development [190,191]. Although supported by strong experimental and mechanistic rationale, anti-virulence therapies remain largely confined to preclinical research. To date, clinical translation has been limited, and no anti-virulence agents are recommended for routine clinical use in the current ESCMID or IDSA guidelines, underscoring the need for robust clinical trials to define their therapeutic relevance.

Precision antimicrobial strategies, supported by rapid molecular diagnostics, pathogen-specific profiling, and resistance gene detection, are increasingly recognized as central components of future clinical practice. Rapid diagnostic tools enable the early identification of causative pathogens and resistance mechanisms, facilitating tailored antimicrobial therapy, improving clinical outcomes, and supporting antimicrobial stewardship by minimizing unnecessary broad-spectrum antibiotic use [192,193]. Both the ESCMID and IDSA guidelines strongly endorse the integration of rapid diagnostics into stewardship programs, particularly in the management of sepsis and bloodstream infections. However, widespread implementation remains constrained by costs, infrastructure requirements, limited availability, and challenges in integrating these technologies into routine clinical workflows.

Overall, while these emerging strategies offer promising alternatives or adjuncts to traditional antibiotic therapy, their integration into clinical practice requires further high-quality evidence, protocol standardization, and the careful evaluation of safety, feasibility, and cost-effectiveness. Future research should focus on personalized, pathogen-targeted approaches that align innovative therapeutic modalities with established antimicrobial stewardship principles, particularly in the management of severe infections such as intra-abdominal infections and sepsis.

## 7. Conclusions and Perspectives

*E. coli* occupies a uniquely plastic niche within the human gastrointestinal tract, where it can function as a benign commensal, a mutualistic symbiont, or under defined ecological and host-specific pressures, a context-dependent contributor to chronic intestinal pathology. In IBD, converging evidence indicates that particular phylogenetic lineages, most notably AIEC, exhibit functional reprogramming or selective enrichment that enables them to exploit the inflamed mucosal environment. This transition is not attributable to a single virulence determinant but instead reflects a coordinated interplay between microbial genomic adaptability, host immunogenetic background, microbiome dysbiosis, and selective pressures imposed by antibiotic exposure.

Central to this process are the pathophysiological and immune interactions that govern the dialogue between *E. coli* and the host. Pathogenic strains display enhanced epithelial tropism mediated by allelic variants of *FimH* and other adhesins, facilitating invasion and intracellular persistence. Once internalized, these strains subvert autophagy and endolysosomal maturation, processes frequently impaired in individuals carrying IBD-associated variants in *NOD2*, *ATG16L1*, or *IRGM*. Their survival within macrophages and dendritic cells drives sustained activation of the NF-κB, STAT3, and MAPK pathways, amplifying the secretion of IL-6, IL-8, TNF-α, and other mediators that perpetuate mucosal inflammation. The presence of genotoxic elements such as the *pks* island, encoding colibactin, links *E. coli* dysbiosis to epithelial DNA damage, cellular senescence, and potentially colorectal carcinogenesis, underscoring the organism’s capacity to influence long-term disease trajectories.

The expanding resistome of intestinal *E. coli* adds a further layer of complexity. Recurrent antibiotic exposure, common in IBD management, disrupts colonization resistance and selects for multidrug-resistant strains with enhanced ecological fitness in the inflamed gut. Resistance plasmids can co-localize with virulence loci, facilitating horizontal gene transfer and accelerating the emergence of pathobiont phenotypes. Thus, antibiotic resistance functions not merely as a therapeutic challenge, but as an evolutionary force that shapes *E. coli* persistence, competitiveness, and pathogenic potential.

Parallel advances in diagnostic methodologies have begun to illuminate the strain-level heterogeneity and functional diversity of *E. coli* populations in IBD. High-resolution molecular tools, including targeted PCR panels, whole-genome sequencing, metagenomics, and metatranscriptomics enable the precise identification of virulence determinants, metabolic signatures, and resistance genes. Complementary phenotypic assays, such as epithelial adhesion/invasion models and macrophage survival assays, provide functional validation of AIEC-like behavior.

Integrative multi-omic approaches that combine microbial genomics with host transcriptomic, metabolomic, and immunologic profiling hold promise for developing clinically actionable biomarkers capable of predicting pathogenic colonization, disease activity, or therapeutic response. However, the absence of standardized criteria for defining AIEC and related pathobionts remains a major impediment to clinical translation. AIEC is increasingly recognized as a pathobiont associated with disease progression, supported by robust experimental evidence; the establishment of direct causality between AIEC and human IBD requires further longitudinal and mechanistic studies.

Collectively, these insights underscore the need for mechanistically grounded, longitudinal, and systems-level frameworks to disentangle causality from correlation. Understanding how host genetics, immune tone, diet, microbial ecology, and antibiotic exposure converge to modulate *E. coli* behavior will be essential for establishing whether these strains act as initiators, amplifiers, or opportunistic responders in IBD pathogenesis.

Such knowledge is essential for the rational design of next-generation therapeutic strategies aimed not at indiscriminate bacterial eradication in selected patient subsets, but at the selective modulation of pathogenic *E. coli* functions while preserving beneficial commensal populations. Promising avenues include bacteriophage-based therapies capable of targeting specific *E. coli* lineages, CRISPR-mediated approaches for strain-level editing or decolonization, engineered probiotics and defined microbial consortia designed to restore colonization resistance, metabolic niche modulation to limit pathobiont competitiveness, and host-directed or immunomodulatory interventions that attenuate inflammation-dependent bacterial persistence.

Ultimately, resolving the multifaceted role of *E. coli* in chronic intestinal disease will require coordinated efforts that integrate multi-omic analyses, ecological modeling, advanced *in vitro* and *in vivo* systems, and longitudinal human cohorts. These interdisciplinary approaches will be indispensable for translating mechanistic insights into precision diagnostic and therapeutic strategies capable of addressing the complex and dynamic interplay between *E. coli*, the host immune system, and the intestinal ecosystem.

## Figures and Tables

**Table 1:** *E. coli* virulence factors specifically associated with inflammatory bowel disease (IBD).

*E. coli* Group (Pathotype, Lineage, or Functional Phenotype)	IBD Association	Key Virulence Factors & Mechanisms (Validated)	Type of Experimental Evidence	Key References
AIEC	CD (ileal)	-Type 1 pili-FimH variants: High-affinity binding to CEACAM6, overexpressed on inflamed ileal epithelium. -Epithelial invasion: FimH-dependent internalization via actin remodeling and macropinocytosis. -Intramacrophage survival: Replication within macrophages with sustained TNF-α production and resistance to apoptosis.	Primary experimental research: -*in vitro* (intestinal epithelial cells) -*in vivo* (CEACAM-transgenic mice) -isogenic mutant analysis	[54,55]
DAEC	UC (pathobiont)	-Afa/Dr adhesins: Binding to DAF (CD55) and CEACAMs, inducing receptor clustering. -Epithelial barrier injury: Microvilli effacement, cytoskeletal rearrangements, and junctional disruption. -Inflammatory signaling: Pro-inflammatory cytokine induction; proposed role as *silent pathobiont*.	Primary experimental research (historic): *in vitro* (polarized intestinal epithelial cell models)	[56,57]
EAEC	CD and UC (pathobiont)	-Aggregative Adherence Fimbriae (AAF): “Stacked-brick” adherence and biofilm formation. -Pic serine protease: Mucinase activity degrading the mucus barrier. -Barrier dysfunction: Tight-junction disorganization and IL-8 induction, promoting chronic inflammation.	Primary experimental research: -*ex vivo* (human intestinal organoids) -*in vitro* -structural biology	[53,58]
B2/D ExPEC-like *E.coli* (pks^+^ lineages)	CD and UC increased CRC risk in long-standing IBD	-*pks* genomic island/colibactin: Genotoxin causing DNA double-strand breaks and characteristic colorectal cancer–associated mutational signatures. -Adhesin-dependent epithelial binding: Type 1 pili (FimH) are required to achieve close host–bacterium contact and enable colibactin-mediated genotoxicity. -Iron/heme acquisition systems (e.g., ChuA): Contribute to bacterial fitness and persistence in the inflamed intestinal niche.	Primary experimental research: -*in vivo* (CRC-prone murine models) -whole-genome sequencing of clinical isolates -pharmacological inhibition of bacterial adhesion	[51,59,60]

The boundaries between *Escherichia coli* pathotypes and phylogroups are fluid, and individual strains may exhibit overlapping features depending on genetic background, host environment, and inflammatory context. The table summarizes virulence factors and functional traits implicated in the pathogenic mechanisms of *E. coli* strain groups associated with IBD, together with the type of experimental evidence supporting these associations. Strain groups are presented as functional, pathotype-based, or genomically defined frameworks, and listed virulence factors represent experimentally validated traits frequently associated with specific pathological contexts, rather than universal or defining markers. AIEC: adherent-invasive *E. coli* (functional phenotype); DAEC: diffusely adherent *E. coli* (Afa/Dr^+^); EAEC: enteroaggregative *E. coli*; B2/D ExPEC-like: genomically defined *pks^+^ E. coli* lineages.

**Table 2:** Plasmids from antibiotic-resistant *E. coli*.

Plasmid Family	Associated Resistance Genes	Main Functions	Transferability to Other Microorganisms (e.g., *Klebsiella pneumoniae*)
IncF	*blaTEM*, *blaCTX-M*, *aadA*, *strA/strB*, *tetA*	Extended-spectrum β-lactamases (ESBL), aminoglycoside and tetracycline resistance; highly prevalent in AIEC strains	High—conjugative plasmids readily transferable across *Enterobacteriaceae*
IncI1	*qnrS*, *qnrB*, *blaCTX-M-1/15*, *sul1/sul2*	Resistance to quinolones, ESBL, sulfonamides	High—efficiently transferred to *Klebsiella*, *Salmonella*, *Shigella*
IncN	*qnr*, *dfrA*, ESBL	Quinolone and trimethoprim resistance; highly mobile plasmids	Very high—known for rapid interspecies dissemination
IncX (X1, X3, X4)	*mcr-1*, *blaNDM*	Colistin and carbapenem resistance	Very high—major drivers of global *mcr-1* spread between *E. coli* and *Klebsiella*
IncHI2	*mcr-1*, ESBL, heavy-metal resistance genes	Multidrug resistance; intestinal adaptation	High—transferable to many *Enterobacteriaceae*, including *Klebsiella*
ColE-type	*qnr*, *aac(6′)*, *fosA*	Small non-conjugative but mobilizable plasmids	Moderate—require helper plasmids for mobilization

Identification of antibiotic-resistant *E. coli* plasmids in the colonic tissues/stools of patients with IBD [86,87].

## Data Availability

No new data were created or analyzed in this study.

## Abbreviations

The following abbreviations are used in this manuscript:

aEPEC	Atypical enteropathogenic *E. coli*
AIEC	Adherent-invasive *E. coli*
AMR	Antimicrobial resistance
ArcA/ArcB	Membrane bound sensor kinase/response regulator
ARG	Antibiotic resistance gene
ASCA	Anti-saccharomyces cerevisiae antibody
ATG16L1	Autophagy related 16 like 1
bo_3_	Cytochrome ubiquinol oxidase
CARD15	Caspase recruitment domain-containing protein 15
CD	Crohn’s disease
CEA	Carcinoembryogenic antigen
CEACAM	Carcinoembryonic antigen-related cell adhesion
CRC	Colorectal cancer
CRISPRi	Clustered regularly interspaced short palindromic repeats interference
CX3CR1	CX3C motif chemokine receptor 1
DAEC	Diffusely adherent E.coli
DEC	Diarrheagenic *E. coli*
DNA	Deoxyribonucleic acid
EAEC	Enteroaggregative *E. coli*
EHEC	Enterohemorrhagic *E. coli*
EIM	Extraintestinal manifestations
EMB	Eosin methylene blue
EPEC	Enteropathogenic *E. coli*
ESBL	Extended spectrum β lactamases
ETEC	Enterotoxigenic *E. coli*
ExPEC	Extra-intestinal pathogens *E. coli*
hDAF	Human decay-accelerating factor
HIF-1α	Hypoxia-inducible factor 1 alfa
IBD	Inflammatory bowel disease
IFN-β	Interferon-beta
IFN-γ	Interferon-gamma
IL-10	Interleukin-10
IL-12	Interleukin-12
IL-17	Interleukin-17
IL-18	Interleukin-18
IL-23	Interleukin-23
ILC1	Group 1 innate lymphoid cells
ILC3	Innate lymphoid type 3 cell
IRGM	Immunity related GTPase M
LAMP	Loop-mediated isothermal amplification
LPS	Lipopolysaccharides
MAPK	Mitogen-activated protein kinases
MIC	Minimum inhibitory concentration
MMR	Mismatch repair
MNPs	Mononuclear phagocytes
mRNA	Messenger ribonucleic acid
mtROS	Mitochondrial reactive oxygen species
NADH	Nicotinamide adenine dinucleotide phosphate
NF-κB	Nuclear factor kappa-light-chain-enhancer of activated B cells
NK	Natural killer cells
NOD2	Nucleotide-binding oligomerization domain-containing protein 2
OM	Outer membrane
OmpA	Outer membrane protein A
OxyR	Oxidative stress regulator
pANCA	Perinuclear antineutrophil cytoplasmic antibody
PFGE	Pulsed-field gel electrophoresis
PKS–NRPS	Polyketide synthase-non-ribosomal peptide synthetase
PPAR-γ	Peroxisome proliferator-activated receptor gamma
PRRs	Pattern recognition receptors
ROS	Reactive oxygen species
SAT	Secreted autotransporter toxin
SoxRS	Superoxide oxidation (o response) sensor/regulator
STAT3	Signal transducer and activator of transcription 3
STEC	Shiga toxin-producing *E. coli*
T3SS	Type III secretion system
Th1	T helper 1 cell
Th17	T helper 17 cell
UC	Ulcerative colitis
UTI	Urinary tract infection
